# Editorial: Interpersonal synchrony and network dynamics in social interaction, volume II

**DOI:** 10.3389/fnhum.2025.1667942

**Published:** 2025-08-01

**Authors:** Viktor Müller, Merle T. Fairhurst, Floris T. van Vugt, Peter E. Keller

**Affiliations:** ^1^Max Planck Institute for Human Development, Center for Lifespan Psychology, Berlin, Germany; ^2^Center for Tactile Internet with Human-in-the-Loop (CeTI), TU Dresden, Dresden, Germany; ^3^Department of Psychology, University of Montreal, Montreal, QC, Canada; ^4^Department of Clinical Medicine, Center for Music in the Brain, Aarhus University, Aarhus, Denmark; ^5^MARCS Institute for Brain, Behaviour and Development, Western Sydney University, Penrith, NSW, Australia

**Keywords:** interpersonal action coordination, hyperscanning, intra-and inter-brain synchronization, network topology and network dynamics, functional connectivity, group interaction

Human beings are capable of remarkable feats of collaboration—ranging from performing complex symphonic music to constructing pyramids. Yet, the precise mechanisms that enable such finely tuned coordination, particularly the neural processes that support it, remain largely elusive. This Research Topic (Volume II) was launched with the aim of further highlighting and exploring the mechanisms and functions of interpersonal interaction, and of deepening our understanding of these highly interesting and complex phenomena and their downstream effects on real-life social interaction. The Research Topic of contributions includes four Perspective and four Original Research articles written by leading researchers in the field. They showcase the breadth of research studies, going from inter-brain synchronization, desynchronization, and causality, hyper-brain network topology, the representation of self and others in emotional contagion and joint action, effects of individual practice, dynamic embodiment, and sync with oneself to pleasantness in joint musical synchronization. This range exemplifies the promise of this field in being able to span multiple facets of life and social dynamic interaction. The research united under the present Topic also illustrates how theoretical insights go hand in hand with advances in methodological tools, enabling increasingly refined empirical investigations.

Notably, several theoretical advances have been proposed as part of this Research Topic. A recent perspective in social and cognitive neuroscience suggests that interpersonal action coordination and communicative behavior depend on inter-brain synchronization and specific hyper-brain network activity, as studied through hyperscanning methods. A Perspective Article by Froese et al. explores the role of *inter-brain desynchronization* (IBD) in social interaction, proposing that it complements the established concept of inter-brain synchrony (IBS) in human neuroscience. Hyperscanning approaches, which study neural dynamics during social interactions, have largely focused on IBS, the synchronization of brain activity between interacting individuals. However, the results have been inconsistent, prompting a re-evaluation of the field's theoretical underpinnings. The authors introduce *irruption theory* (Froese, 2023), which suggests that subjective involvement in social interactions increases neural entropy, leading to IBD, or decreased phase synchrony between brains. This desynchronization may act as a counterbalance to IBS, explaining the observed variability in IBS findings. While IBS has been considered the main marker of social engagement, irruption theory posits that IBD may play a positive role by enabling cognitive flexibility and adaptation in dynamic social contexts, such as turn-taking or complementary behaviors. Potential biases are highlighted within the field that have prioritized IBS over IBD, suggesting that the lack of evidence for IBD may be due to this narrow focus. The authors advocate for broader consideration of IBD, which they argue could provide a more nuanced understanding of social coordination. Moreover, they suggest that the interplay between IBS and IBD, rather than the dominance of one, may be crucial for healthy social interactions and mental wellbeing. Future research should systematically examine the variability of IBS and explore how IBS and IBD together contribute to the dynamic nature of human social behavior.

Emotional contagion is the process by which an emotional state is transferred from one individual to another, describing the spread and mutual influence of emotions among individuals. In contrast, social buffering occurs when an individual's stress response is diminished due to the presence of one or more others. While each process has been extensively studied independently, the relationship between them remains unclear (Reimert and Bolhuis, 2024). A Perspective article by Wang et al. explores the complex interaction between social context, self-representation, and emotional contagion, challenging the notion that emotional contagion occurs automatically and unconsciously. Instead, the authors propose that self-representation—how individuals perceive themselves within a social context—plays a crucial role in shaping emotional contagion. The study highlights how social settings activate specific self-representations, such as one's identity or role, which influence how individuals emotionally respond to others. For instance, when people are aware of their social roles or relationships in a group, their self-representation becomes contextualized, affecting their susceptibility to emotional contagion. The authors introduce a dynamic model that describes a causal loop: social contexts activate self-representation, which then influences emotional contagion, and, in turn, emotional contagion strengthens interdependent self-representation, fostering social connection. This model emphasizes the bidirectional relationship between emotional contagion and social dynamics. The authors also highlight the need to study emotional contagion in group settings and organizational contexts, as well as the role of cultural factors in influencing the relationship between self-representation, social context, and emotional contagion, providing a broader perspective on its impact across different environments.

Throughout various epochs and cultures, humans have come together to make music collectively—a universal behavior that remains both intriguing and only partially understood. As interest in joint music-making continues to grow, recent advancements in this emerging field, integrating insights from behavioral, neural, and computational research, are exciting (Keller, 2023; Abalde et al., 2024). An Original Research article by Plitchenko et al. investigates how individual practice impacts joint synchronization during musical performances, focusing on sensorimotor synchronization in solo and ensemble settings. Musically trained participants practiced producing a melody by tapping on a keypad, receiving either normal or delayed auditory feedback. The study examined how these solo practices influenced their synchronization when performing as duets. Results showed that synchronization accuracy was higher during joint performances than in solo practices, even though solo tasks involved more temporally regular cues. Interestingly, participants' ability to synchronize improved over time, indicating a learning effect. Delayed auditory feedback in solo conditions increased asynchrony, but when participants practiced with normal feedback, they performed better during subsequent joint tasks. The study also applied a delay-coupling oscillator model, revealing that coupling strength between partners was stronger after normal feedback practice compared to delayed feedback. The findings suggest that individual practice, particularly with accurate feedback, enhances synchronization in duet performances. This supports the effectiveness of solo practice methods commonly used by musicians to prepare for ensemble performances. The study also highlights that joint synchronization benefits from the inherent variability of a partner's timing, which musicians can adapt to more easily than a computer-generated rhythm.

Humans interact through actions mediated by sensory and motor processes, with intra- and inter-brain synchrony oscillations supporting social adjustment. However, it remains unclear whether IBS can be attributed to similar bottom–up processes during synchronous play, or if it instead reflects cognitive top-down control required for periods of higher coordination demands (Müller et al., 2021; Gugnowska et al., 2022; Lender et al., 2023). Varlet and Grootswagers examine the limitations of current hyperscanning research, particularly regarding IBS as a measure of social interaction. Hyperscanning allows for simultaneous brain activity recording from multiple individuals and has been used to explore how brain waves align during social interaction. However, the authors argue that IBS, often used as a proxy for synchronized cognitive and sensory processes, is not sensitive enough to capture interpersonal information alignment, echoing theoretical proposals such as irruption theory (Froese, 2023). Through EEG hyperscanning simulations, they show that IBS remains largely unchanged even when two individuals are exposed to different visual stimuli simultaneously. This suggests that IBS reflects timing alignment rather than the content of sensory and cognitive processes. The study challenges the notion that IBS directly causes synchronized minds and behaviors. Instead, it supports the view that synchronized brain activity is a byproduct of coordinated behavior and cognition, not the driving force behind it. The authors also highlight discrepancies in past hyperscanning studies, where IBS was not consistently observed, even when participants were engaged in social interaction. To address these issues, the article proposes using representational analyses as a more effective method for capturing interpersonal information alignment. Unlike IBS, representational analyses allow for the comparison of information content across individuals, making them a promising alternative for future research. The authors call for further development of these methods, especially for more naturalistic and complex social tasks, to enhance our understanding of the neuropsychological processes underlying real-time social interactions.

Neural cell assemblies emerging within interacting brains require continuous adjustment and close coordination to support interpersonal dynamics and interactive activities that often operate on millisecond time scales. A *hyper-brain cell assembly* was hypothesized to integrate oscillatory activity both within and between brains, as well as across different oscillation frequencies, forming a unified structure responsible for social and interactive behavior (Müller, 2022). An article by Müller and Lindenberger explores the neural dynamics of ensemble music performance by examining how different brain oscillations (neural frequencies) interact within and between the brains of musicians in a guitar quartet. Using EEG data, the study focuses on cross- and within-frequency coupling (WFC and CFC, respectively) to construct hyper-brain hyper-frequency networks (HB-HFNs), a multilayer network organization, providing insights into how neural coordination supports synchronized musical performance. The findings suggest that low-frequency oscillations (such as delta, theta, and alpha waves) play an integrative role in coordinating actions between musicians, with each guitarist contributing uniquely to the network dynamics. Notably, coupling strength decreases with higher oscillation frequencies. Additionally, the study shows that WFC is generally stronger within individual brains, while CFC is more prominent between brains. The topology of HB-HFNs appears influenced by musical acoustic properties, such as amplitude and frequency. The complex multilayer network structures are also found to be robust against the loss of connections, particularly when the strongest connections are maintained. The authors conclude that HB-HFNs effectively capture the neural processes involved in coordinating interpersonal actions during ensemble performance. The findings highlight how multilayer brain networks dynamically integrate sensory and motor information to support synchronized group behavior. This study extends previous research on neural markers of interpersonal action coordination, particularly in complex, real-time social activities like music performance, and offers a versatile framework for studying neural interactions in various forms of social behavior.

Music-making is a universal process of creating aesthetically pleasing sound patterns through interpersonal synchronization, involving the coordination of actions, emotions, thoughts, and physiological rhythms, yet its impact on the sensorimotor interactions between participants remains unclear. An article by Lazzari et al. investigates how the aesthetic quality of jointly produced sounds influences interpersonal motor coordination. Using a dyadic synchronization-continuation task (dSCT), non-musician pairs tapped in synchrony to a metronome before continuing at the same tempo without it. Each tap generated a note, creating either consonant (pleasurable) or dissonant (less pleasurable) chords. Results showed that dyads synchronized more closely when producing consonant chords and rated these interactions as more enjoyable. Interestingly, consonance affected synchronization only in the joint continuation phase, not in individual metronome-paced tapping. Furthermore, the synchronization effect of consonant sounds was more pronounced in pairs who initially felt less socially close, suggesting that consonance enhances both coordination and social connection. The findings highlight the role of aesthetic harmony in shaping social and motor synchrony. Beyond academic interest, the results hold potential clinical applications. For example, consonant musical intervals could improve motor synchronization in therapeutic settings, particularly for individuals with sensory-motor deficits, such as those in Parkinson's disease or schizophrenia. Synchronizing to consonant sounds might foster movement coordination, enhance social connection, and improve therapeutic outcomes. This research emphasizes that the aesthetic outcomes of joint activities can have tangible benefits for both movement precision and interpersonal rapport.

Inter-brain coupling is investigated as a predictor of behavioral change, but despite advances in hyperscanning that illuminate its role in social interactions, establishing causal links between brain activity and behavior remains a significant challenge. A Perspective article by Markus and Shamay-Tsoory delves into Research Topic, offering potential methodologies and valuable insights into how inter-brain coupling underpins essential processes in social interactions. While current research has correlated inter-brain coupling with changes in social interaction, proving causation remains difficult due to differing timescales of neural and behavioral responses and a lack of causal-focused methods. To address this, the authors propose two approaches: dyadic neurofeedback, which reinforces inter-brain synchrony to observe whether such coupling affects mutual synchronization attempts, and statistical techniques like Granger causality and Structural Equation Modeling (SEM). Granger causality allows for predicting how neural interactions might drive behavior, while SEM provides a detailed framework for modeling direct and indirect influences of neural synchrony on behavior. The article highlights the need for more robust causal methods within a network model of social interaction, suggesting that Granger causality and SEM could allow researchers to discern directional influences between brain coupling and behavior. The authors propose an expanded model—multilevel SEM (mSEM)—that could integrate multiple behavioral and neural components, enabling a comprehensive view of social interaction dynamics. By testing causality with these methods, future studies could clarify how neural synchrony contributes to social behaviors, moving beyond mere correlation to identify specific causal pathways in social cognition and interaction.

Understanding how groups of people coordinate movement in rhythmic settings remains a central challenge in research on social interaction and collective behavior. Toiviainen et al. tackle this by presenting an innovative modeling approach that captures the dynamics of dance floor behavior. Extending traditional swarmalator models, the authors introduce “directional swarmalators,” integrating gaze direction alongside spatial movement and rhythmic synchronization. This addition allows their model to better simulate how dancers dynamically self-organize based on both musical and visual cues. Validated against motion capture data from silent disco experiments, the model captures emergent patterns such as circular group formations and highlights the importance of visual attention in collective coordination. Despite some instructive limitations—such as overly smooth agent movements and the need for broader datasets—the model offers a powerful tool for studying large-group social dynamics, with potential applications beyond dance. The authors suggest that future developments could include simulating more naturalistic erratic movement, expanding to other collective behaviors, and integrating anticipatory processes crucial for human synchronization. Overall, this work offers a compelling framework for linking visual perception, rhythmic behavior, and emergent group structures in dynamic environments.

Understanding how individuals coordinate actions in real time is central to studying social interaction, particularly in complex activities like ensemble music performance. Kohler et al. address this by examining the neural representations underlying self- and other-produced actions in duetting pianists. Using multivariate pattern analysis (MVPA) of fMRI data, the study demonstrates that expert pianists maintain parallel, distinct, and content-specific neural representations for their own and their partner's musical parts while playing duets. Remarkably, primary motor cortex (M1) primarily encoded self-produced actions, while premotor cortex (PMC) encoded the partner's actions, with an unexpected lateralization pattern: left M1 for self and right PMC for other. These findings challenge existing notions about hemispheric specialization and provide empirical support for theories positing separate yet integrated internal forward models facilitating interpersonal coordination. Interestingly, the precision of motor representations was not strongly dependent on prior motor familiarity with the partner's part, suggesting that auditory-motor expertise alone may support flexible coordination with novel musical material. By combining univariate and multivariate neuroimaging approaches, the study opens new avenues for understanding the neural architecture of *social action prediction* and *self-other integration* in music and beyond, highlighting how sophisticated internal models guide joint performance even without explicit motor rehearsal.

The coordination of simultaneous actions within a single individual—spontaneous intrapersonal coordination—is an emerging topic that bridges motor control, cognitive load, and rhythm research. Jagadeesan and Grahn investigate how different types of periodic behaviors, such as finger tapping, walking, and vocalizing, coordinate within individuals under varying cognitive demands. Across two experiments, the authors show that simultaneous periodic actions exhibit higher coordination than when performed separately, but this coordination is sensitive to the nature of the task and the presence of additional cognitive load. Coordination between tapping and vocalizing was more stable than between tapping and walking, suggesting that walking imposes greater cognitive demands, likely due to its complexity. Moreover, adding a concurrent cognitive task—such as backward counting or visual pattern matching—reduced coordination stability, particularly for tapping and walking combinations. These findings position spontaneous intrapersonal coordination as a promising avenue for understanding how attentional and cognitive resources modulate motor behavior. They also suggest that coordination stability could serve as a sensitive marker of cognitive load. Overall, the study highlights the intricate interplay between cognition and action and opens new pathways for examining how the brain manages multiple rhythmic activities simultaneously.

The studies presented in this Research Topic showcase a remarkable breadth of themes and research questions, underscoring the significance of interpersonal action coordination and social interaction across a range of contexts. Collectively, they reinforce the view that neuronal dynamics underlying social interaction are not confined to isolated brain regions but emerge from system-wide processes involving synchronized activity across brains and sensorimotor systems. This inter-brain and inter-system synchrony, which emerges across varying contexts and situations, reflects the inherently complex and distributed nature of human interaction. Importantly, the findings open promising avenues for both fundamental research and clinical applications—particularly in domains such as neurorehabilitation, social cognition, and communication disorders. Furthermore, these contributions demonstrate the growing relevance of hyperscanning methodologies in advancing social neuroscience, offering a powerful framework for understanding how shared neural processes support real-time coordination between individuals.

## References

[B1] Abalde S. F. Rigby A. Keller P. E. Novembre G. (2024). A framework for joint music making: behavioral findings, neural processes, and computational models. Neurosci. Biobehav. Rev. 167:105816. 10.1016/j.neubiorev.2024.105816 39032841

[B2] Froese T. (2023). Irruption theory: a novel conceptualization of the enactive account of motivated activity. Entropy 25:748. 10.3390/e25050748 37238503 PMC10217218

[B3] Gugnowska K. Novembre G. Kohler N. Villringer A. Keller P. E. Sammler D.. (2022). Endogenous sources of interbrain synchrony in duetting pianists. Cerebral Cortex 32, 4110–4127. 10.1093/cercor/bhab469 35029645 PMC9476614

[B4] Keller P. E. (2023). Integrating theory and models of musical group interaction. Trends Cogn. Sci. 27, 1105–1106. 10.1016/j.tics.2023.07.008 37739920

[B5] Lender A. Perdikis D. Gruber W. Lindenberger U. Müller V. (2023). Dynamics in interbrain synchronization while playing a piano duet. Ann. N. Y. Acad. Sci. 1530, 124–137. 10.1111/nyas.15072 37824090

[B6] Müller V. (2022). Neural synchrony and network dynamics in social interaction: a hyper-brain cell assembly hypothesis. Front. Hum. Neurosci. 16:848026. 10.3389/fnhum.2022.848026 35572007 PMC9101304

[B7] Müller V. Ohström K-. R. P. Lindenberger U. (2021). Interactive brains, social minds: neural and physiological mechanisms of interpersonal action coordination. Neurosci. Biobehav, Rev. 128, 661–677. 10.1016/j.neubiorev.2021.07.017 34273378

[B8] Reimert I. Bolhuis J. E. (2024). Possible relations between emotional contagion and social buffering. Sci. Rep. 14:25944. 10.1038/s41598-024-77394-7 39472724 PMC11522516

